# *Bacteroides fragilis* Toxin Induces Sequential Proteolysis of E-Cadherin and Inflammatory Response in Mouse Intestinal Epithelial Cell Line

**DOI:** 10.3390/microorganisms13040781

**Published:** 2025-03-28

**Authors:** Woo-Seung Kim, Soonjae Hwang, Sun-Yeong Gwon, Minjeong Jo, Sang-Hyeon Yoo, Jiyun Hong, Ha-Neul Jang, Ju-Eun Hong, Da-Hye Kang, Miyong Yun, Ki-Jong Rhee

**Affiliations:** 1 Department of Biomedical Laboratory Science, College of Software Digital Healthcare Convergence, Yonsei University at MIRAE Campus, Wonju 26493, Republic of Korea; redberry1245@yonsei.ac.kr (W.-S.K.); yshyyb@yonsei.ac.kr (S.-H.Y.); hongjy7pc@yonsei.ac.kr (J.H.); zzhaneul0@yonsei.ac.kr (H.-N.J.); jehong@yonsei.ac.kr (J.-E.H.); 2 Department of Biochemistry, Lee Gil Ya Cancer and Diabetes Institute, College of Medicine, Gachon University, 155 Gaetbeol-ro, Inchon 21999, Republic of Korea; soonjae@gachon.ac.kr; 3 Neural Circuits Research Group, Korea Brain Research Institute, Daegu 41062, Republic of Korea; sunyeong.gwon@kbri.re.kr; 4 Department of Structural Biology, St. Jude Children’s Research Hospital, Memphis, TN 38105, USA; minjeongjo12@gmail.com; 5 Department of Obstetrics, Gynecology and Women’s Health, University of Missouri, Columbia, MO 65211, USA; dahyekang@missouri.edu; 6 Department of Bioindustry and Bioresource Engineering, College of Life Sciences, Sejong University, Seoul 05006, Republic of Korea

**Keywords:** *Bacteroides fragilis* toxin, mouse epithelial cell, E-cadherin, KC, LIX, sTNFR1

## Abstract

Enterotoxigenic *Bacteroides fragilis* (ETBF) is an intestinal bacterium that secretes the metalloprotease *Bacteroides fragilis* toxin (BFT), which induces E-cadherin cleavage and interleukin-8 secretion in human intestinal epithelial cell lines. ETBF-induced E-cadherin cleavage is proposed to be the underlying reason for the promotion of colitis in ETBF-infected mice. However, a BFT-responsive murine cell line has not yet been reported. In the current study, we report that the mouse colonic epithelial cell line CMT93 undergoes E-cadherin ectodomain cleavage, cell rounding, and proliferation in response to BFT treatment. The amino acid sequence of the putative cleavage site of E-cadherin is identical in both BFT-responsive (CMT93) and BFT-nonresponsive (MSIE, CT26, YAMC, and B16) cell lines, suggesting that the E-cadherin amino acid sequence is not responsible for this observation. After E-cadherin ectodomain cleavage, the membrane-bound intracellular E-cadherin domain underwent cleavage by γ-secretase and was subsequently degraded by the proteasome. Moreover, BFT induced the secretion of two chemokines (LIX and KC) and the formation of soluble TNFR1 in the CMT93 cell line. The identification of a BFT-responsive murine cell line may be used to elucidate the mechanism of ETBF pathogenesis in ETBF murine infection models.

## 1. Introduction

*Bacteroides fragilis* is a colonic commensal bacterium that colonizes the majority of humans [1]. Despite being a minority (~0.1%) of the total fecal flora, *B. fragilis* is a major etiologic anaerobic pathogen causing bacteremia and intraabdominal abscesses [2,3]. A subset of *B. fragilis* strains, termed enterotoxigenic *B. fragilis* (ETBF), is associated with the onset of diarrheal disease in both humans and animals, as well as the promotion of inflammatory bowel disease (IBD) in humans [4,5]. It has been reported that ETBF contains a pathogenicity island that is involved in bacterial virulence and strain competition [6,7]. This pathogenicity island possesses the *B. fragilis* toxin (BFT) gene (*bft*), whereas strains of *B. fragilis* lacking the *bft* gene are called non-toxigenic *Bacteroides fragilis* (NTBF) [8]. BFT biological activity in intestinal epithelial cells are mitigated by the mutation of the catalytic motif [9,10]. In vitro, the human colonic epithelial cell line HT29/c1 and in vivo mouse infection studies clearly showed that point mutations within the BFT catalytic domain resulted in the complete loss of BFT biological activity [9,11].

The most well-known biological activity of BFT is the induction of E-cadherin cleavage in HT29/c1 cells. E-cadherin is a 120 kDa glycosylated type I transmembrane protein, which is responsible for the formation of the adherence junction between adjacent epithelial cells and essential to proliferation and differentiation [12,13]. In primary human colon cells and colonic epithelial cell lines in vitro, BFT increases the disruption of the intact epithelial barrier due to E-cadherin ectodomain cleavage [14,15]. In addition, the loss of the adherence junction results in cell rounding, cell proliferation, and IL-8 secretion. In BFT-treated HT29/c1 cells, the E-cadherin ectodomain is rapidly (~30 min) cleaved, and the 33 kDa cell-membrane-tethered remnant is further cleaved intracellularly by γ-secretase. The γ-secretase cleavage results in the formation of two fragments, a 28 kDa cytoplasmic fragment and a 5 kDa transmembrane region [16]. Subsequently, the proteasome complex degrades the 28 kDa cytoplasmic fragment [17].

The oral administration of ETBF bacteria in wild-type mice has been used to investigate the effects of BFT in vivo [11]. Similar to BFT-treated HT29/c1 cells, ETBF bacterial infection induces E-cadherin cleavage in the colon in vivo and elicits an inflammatory response by the secretion of keratinocyte chemoattractant (KC) in mouse intestinal epithelial tissues ex vivo [18]. KC is a functional homologue of human IL-8 that acts as a chemoattractant for neutrophils in mice [19]; the human genome does not encode the KC gene. One hallmark of BFT biological activity is the ability to induce IL-8 secretion in BFT-treated HT29/c1 cells [14]. However, due to an absence of a BFT-responsive mouse cell line, it is unknown whether BFT treatment can induce KC secretion in mouse epithelial cells. In mice, LIX (also known as CXCL5) is another cytokine that is involved in activating neutrophils [20]. Similar to KC, the secretion of LIX in BFT-treated mouse cell lines remains to be explored.

ETBF-infected mice demonstrated STAT3 activation and secretion of Th17-related cytokines (IL-23, IL-17,TGF-β, and IL-6) that have a pro-carcinogenic effect on mouse intestine [21]. Although the murine model has been used successfully in understanding ETBF pathogenesis, it is still unclear whether the murine intestinal cells respond to BFT similarly to HT29/c1 cells. Until now, a BFT-responsive cell line has not yet been identified, raising the question whether the mechanistic effects of BFT on human and mouse cells differ. The lack of a BFT-responsive mouse cell line has hindered detailed mechanistic studies at the cellular level. Furthermore, by investigating the differences between BFT-responsive and BFT-nonresponsive mouse epithelial cell lines, the BFT-induced E-cadherin cleavage mechanism may be elucidated. The aim of the current study was to identify a BFT-responsive mouse epithelial cell line and to compare BFT biological activity with the human HT29/c1 cell line.

## 2. Materials and Methods

### 2.1. Cell Culture and BFT Treatment

Cell lines were incubated in Dulbecco’s modified Eagle medium supplemented with 10% fetal bovine serum, 25 mM HEPES, and penicillin (100 U/mL)/streptomycin (100 µg/mL) (Invitrogen, Carlsbad, CA, USA). HT29/c1, CMT93, CT26, and B16 cell lines were obtained from ATCC (Manassas, VA, USA). MSIE and YAMC cell lines were obtained from Robert Whitehead (Ludwig Institute for Cancer Research, Australia). For BFT treatment, cells were washed with serum-free media (SFM) and treated with brain heart infusion broth (BHIB) supernatant cultured with rNTBF or rETBF. Each bacterial supernatant was diluted 20-fold in SFM. For negative control, SFM alone or BHIB that was diluted 20-fold with SFM was used.

### 2.2. Bacterial Strains and Collection of Bacterial Supernatants

Wild-type nontoxigenic *B. fragilis* (WT-NTBF) overexpressing an active form of *bft* (rETBF; *bft*-2) and WT-NTBF overexpressing a biologically inactive mutated *bft* (rNTBF; *bft*-2 H352Y) were generous gifts from Cynthia Sears and Augusto Franco (Johns Hopkins University, Baltimore, MA, USA). Recombinant strains of *B. fragilis* secrete either catalytically active BFT or catalytically inactive BFT. The NTBF strain called *B. fragilis* NCTC 9343 was transformed with the pFD340::*bft*-2 plasmid, allowing for synthesis and secretion of the biologically active BFT. This strain is referred to as recombinant enterotoxigenic *Bacteroides fragilis* (rETBF) [22]. In addition, *B. fragilis* NCTC 9343 was transformed with *bft*-2 H352Y::pFD340, allowing for production and secretion of the biologically inactive BFT. This recombinant strain is referred to as recombinant NTBF (rNTBF) [9]. All *Bacteroides* strains used in this study were naturally resistant to gentamicin and transformed with pFD340, conferring clindamycin resistance. Both recombinant *Bacteroides* strains (rETBF and rNTBF) were grown in brain heart infusion agar (BHIA)(#237500, BD, Sparks, MD, USA) at 37°C under anaerobic conditions (Pack-Anaero, Mitsubishi Gas Chemical Co., Inc., Tokyo, Japan) for two days. BHIA was supplemented with 5 g/L yeast extract, 5 µg/mL hemin, 5 mg/mL L-cysteine, and two antibiotics [6 µg/mL of clindamycin (#C2257, Tokyo Chemical Industry Co., LTD., Tokyo, Japan) and 50 µg/mL of gentamicin (#GA7939, Glentham Life Sciences, Corsham, UK)]. Thereafter, a single bacterial colony was picked and cultured in BHIB at 37 °C under anaerobic condition for another two days. The bacterial supernatants were centrifuged, filtered by 0.45 μm syringe filter (#S6555-FMOSK, Satorius, Goettingen, Germany), and stored at −80 °C until they were used.

### 2.3. Western Blot Analysis

Cells were washed with phosphate buffered saline (PBS) and lysed at 4 °C with radioimmunoprecipitation (RIPA) buffer (#R0278-500ML, Sigma, St. Louis, MO, USA) containing 5% protease inhibitor cocktail (#11836153001, Roche Diagnostics, Mannheim, Germany) and 1% phosphatase inhibitor cocktail (#P5726-1ML, Sigma, St. Louis, MO, USA). The cell lysates were incubated on ice for 10 min and centrifuged at 12,000× *g* for 10 min at 4 °C. The supernatant protein was collected and quantified by Quick Start™ Bradford Protein Assay (Bio-Rad, Hercules, CA, USA). Protein samples were separated using 10% sodium dodecyl sulfate–polyacrylamide gel electrophoresis (SDS-PAGE) and transferred to nitrocellulose membrane (Pall, Washington, NY, USA). For measuring alterations in proteins, membranes were probed with primary antibodies (0.1 μg/mL) overnight at 4 °C. For cell lysates, the following antibodies were used: anti-E-cadherin endodomain-specific monoclonal antibody (clone C36, #610181,BD Transduction Laboratories, Franklin Lakes, NJ, USA), anti-GAPDH monoclonal antibody (#CB1001, Calbiochem, San Diego, CA, USA), or anti-E-cadherin ectodomain-specific polyclonal antibody (H-108, #sc-7870, Santa Cruz, Heidelberg, Germany). The C36 antibody was used to detect the full-length E-cadherin (~120 kDa), the E-cadherin intracellular domain (33 kDa), and the E-cadherin cytoplasmic fragment (28 kDa) in cell lysates. For detecting cleaved E-cadherin ectodomain, supernatants of BFT-treated HT29/c1 and CMT93 cells were concentrated using 10 K Amicon^®^ ultra centrifugal filters (Merck Millipore, Cork, Ireland). The cleaved E-cadherin ectodomain (~80 kDa) fragment was detected using anti-E-cadherin ectodomain-specific antibody (H-108) for 12 h at 4 °C. Horseradish peroxidase (HRP)-conjugated anti-mouse or anti-rabbit IgG (Jackson ImmunoResearch, West Grove, PA, USA) secondary antibodies (0.1 μg/mL) were used to detect primary antibodies for 1 h at room temperature. The immune-labeled proteins were visualized using enhanced chemiluminescence (ECL) kit (#170-5061, Bio-Rad, Hercules, CA, USA). Quantification of relative band intensity was determined using FusionCapt Advance software (version 16.06, Vilber Lourmat, Marne-la-Vallée, France).

### 2.4. Immunofluorescence Staining and Confocal Microscopy

Cells were grown on sterilized glass coverslips and fixed with 4% *p*-formaldehyde solution (BIOSESANG, Seongnam, Republic of Korea) at 4 °C for 10 min. Thereafter, cells were washed with PBS and permeabilized with absolute methanol for 20 min. Regarding immunostaining, cells were blocked with 1% bovine serum albumin (BSA) in PBS for 30 min and stained with a 1 µg of anti-E-cadherin endodomain-specific antibody (C36) in 1% BSA-PBS solution overnight at 4 °C. Then, the cells were labeled with 1:1000 dilution of the Alexa Fluor 488-labeled secondary antibody (Invitrogen, Carlsbad, CA, USA) in 1% BSA-PBS solution at 4 °C for 1 h. Finally, cells were washed three times with PBS and mounted with mounting medium containing 4, 6-diamidino-2-phenylindole (DAPI) (Vector Laboratories, Peterborough, UK). Images were captured with Carl Zeiss LSM710 confocal microscope (Carl Zeiss, Oberkochen, Germany).

### 2.5. RNA Extraction, Reverse Transcription PCR, and DNA Sequencing

Total RNA was isolated from cultured cells using TRIzol^®^ (Invitrogen, Carlsbad, CA, USA) reagent according to the manufacturer’s instruction. Cells were treated with 800 μL of TRIzol^®^ and lysed at RT for 10 min. Then, 200 μL of chloroform was added and centrifuged at 12,000× *g* and 4 °C for 15 min. A volume of 200 μL of the upper transparent layer was transferred to new microcentrifuge tube, and equal amount of isopropanol was added. After 10 min incubation at RT, the mixture was centrifuged at 12,000× *g* and 4 °C for 10 min. Then the supernatant was removed, and 1 mL of 75% ethanol was added. Finally, the mixture was centrifuged at 8500× *g* for 5 min, and the supernatant was removed. The RNA pellet was air-dried and dissolved in 20 μL of diethylpyrocarbonate (DEPC)-treated water (Invitrogen, Carlsbad, CA, USA). RNA quantity and quality were assessed by measuring the optical density at 260 nm and 280 nm using NanoQuant Infinite M200 (Tecan, Männedorf, Switzerland). Complementary DNA (cDNA) was synthesized using 2 μg of total RNA, 0.5 μg of oligo (dT) 12–18 primers (Invitrogen, Carlsbad, CA, USA), and 200 U of M-MLV RT (Invitrogen, Carlsbad, CA, USA) for 50 min at 37 °C and 15 min at 70 °C. Subsequent PCR amplification using 0.2 U of *Taq* polymerase was performed in a TaKaRa thermal cycler dice TP600 (TAKARA, Shiga, Japan). For detecting E-cadherin mRNA expression and sequence, primers overlapping the putative BFT-induced cleavage site, which is between EC5 domain and transmembrane domain of E-cadherin, were designed. Primers for E-cadherin were forward 5′-GTA GCT CTC ATC ATC GCC AC-3′ and reverse 5′-CTC TTT GAC CAC CGT TCT CC-3′. Designed primers were purchased from Cosmogenetech (Seoul, Republic of Korea). PCR conditions were 5 min at 94 °C (initial denaturation); 30 cycles of 30 s at 94 °C (denaturation), 30 s at 55 °C (annealing), and 30 s at 72 °C (extension); and 7 min at 72 °C (final extension). The 588 bp amplicon was confirmed using 2% agarose gel electrophoresis with 1% Tris base, boric acid, and EDTA (TBE) buffer, stained by ethidium bromide (EtBr) for 10 min, and de-stained in distilled water for 20 min. Gel images were taken using Molecular Image Gel DocTM XR+ system (Bio-Rad). For analyzing E-cadherin mRNA sequence, amplified PCR products were purified by AccuPrep^®^ PCR purification kit (Bioneer, Daejeon, Republic of Korea) according to the manufacturer’s instructions. mRNA analysis was conducted by Cosmogenetech (Seoul, Republic of Korea). Amino acid sequences of E-cadherin based on the analyzed mRNA sequences were translated by Snapgene Viewer software (version 6.2.1, Dotmatics, Woburn, MA, USA).

### 2.6. γ-Secretase and Proteasome Inhibitor Study

For γ-secretase inhibitor assay, cells were pre-treated with 1.5 μM γ-secretase inhibitor (L-685,458; Calbiochem, San Diego, CA, USA) in SFM for 1 h and incubated with SFM, rNTBF, or rETBF supernatants. For proteasome inhibitor assay, cells were pre-treated with 2 μM proteasome inhibitor (Bortezomib, #S1013, Selleckchem, Houston, TX, USA) in SFM for 1 h and incubated with SFM, rNTBF, or rETBF supernatants. Inhibitors were added into the cell culture during incubation.

### 2.7. Evaluation of Cellular Proliferation

To enumerate viable cells, HT29/c1 and CMT93 cells were seeded at a density of 5 × 10^4^ cells/well in 60 mm culture plates and cultured for 3 days. Cells were approximately 20% confluent at time of BFT treatment. For BFT treatment, cells were washed with SFM 3 times and treated with BHIB, rNTBF, or rETBF supernatant for 0, 24, 48, and 72 h. After each indicated time point, cells were detached with 0.25% trypsin-EDTA (#25200072, Invitrogen, Carlsbad, CA, USA) stained with trypan blue. Viable cells were counted on a hemocytometer (Marienfeld, Lauda-Königshofen, Germany) under a microscope.

### 2.8. RayBio^®^ Inflammation Antibody Array

The antibody array analysis was conducted as suggested by the manufacturer (#M0328001, RayBiotech, Peachtree Corners, GA, USA). In brief, the antibody array consists of a membrane coated with antibodies specific for 40 different inflammatory mediators. Similar to a “sandwich ELISA” protocol, membranes were blocked for 30 min and then incubated with 2 mL of CMT93 cells’ supernatant for 2 h at room temperature. The unbound proteins were washed, and then an antibody cocktail containing biotinylated antibodies specific for each inflammatory mediator was added to the membrane for 1.5 h. The membranes were washed and then incubated with HRP-conjugated streptavidin for 2 h. The membranes were washed extensively and overlaid with the detection buffer. The chemiluminescence signal emanating from the membranes were exposed to X-ray film. The developed film was scanned, the dot intensity was quantitated, and the results were compared by FusionCapt Advance software (version 16.06, Vilber Lourmat). Three potential targets (KC, LIX, and soluble TNFR1) were found to be elevated in rETBF-treated CMT93 cell culture media compared to rNTBF-treated CMT93 cell culture media. The full list of target inflammatory mediators detectable by this kit is shown in Supplementary Data (Table S1).

### 2.9. Enzyme-Linked Immunosorbent Assay (ELISA) Analysis

For cytokine analysis, cell supernatants were collected and cytokine levels of KC (CXCL1), LIX (CXCL5), and sTNFR1 were determined by ELISA kit (R&D systems, Minneapolis, MN, USA) following the manufacturer’s instruction. In brief, assay diluent was placed into each well of a 96-well plate. Each well was coated with antibodies specific for each cytokine. The supernatant from CMT93 cells was added to each well and incubated for 2 h at room temperature. The unbound proteins were washed with washing buffer 5 times and then incubated with polyclonal antibody conjugated to horseradish peroxidase for 2 h at room temperature. The wells were washed again and then incubated with detection buffer (hydrogen peroxide and chromogen tetramethylbenzidine) for 0.5 h at room temperature. After incubation, stop solution (hydrochloric acid) was added to each well and measured with microplate reader. The intensity of the color was measured at 450 nm using Infinite M200 PRO Multimode Microplate Reader (Tecan).

### 2.10. Statistical Analysis

Data in the bar graphs are presented as mean ± standard error of mean (SEM). All statistical analyses were performed using GraphPad Prism 7.0 software (GraphPad Software, San Diego, CA, USA). All data were analyzed by unpaired *t*-test, and *p* < 0.05 was considered to be statistically significant (* *p* < 0.05, ** *p* < 0.01, and *** *p* < 0.001).

## 3. Results

### 3.1. BFT Induces Loss of E-Cadherin in Murine Epithelial CMT93 Cells

**Figure 1:**
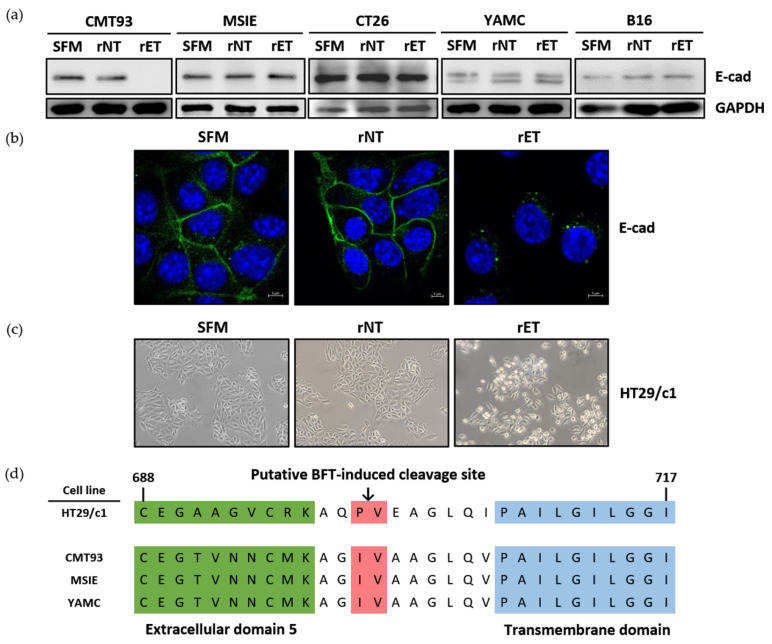
BFT induces E-cadherin cleavage in CMT93 cells. (**a**) Western blot analysis of CMT93, MSIE, CT26, YAMC, and B16 cells treated with serum-free media (SFM), rNTBF, or rETBF supernatant (1:20 dilution) for 3 h. E-cadherin was detected using monoclonal Ab specific for the endodomain of E-cadherin (clone C36). GAPDH, internal control; (**b**) Immunofluorescence imaging of CMT93 cells 3 h post-treatment. E-cadherin (green, clone C36) and nuclei (blue, DAPI) were observed with a confocal laser-scanning microscope (×1600); (**c**) Light microscope images of CMT93 cells observed under an inverted microscope (×40) 3 h post-treatment; (**d**) Putative BFT-induced cleavage site (arrow, red region) in human and mouse epithelial cell lines. Portions of the extracellular domain (green) and the transmembrane domain (blue) are shown. Amino acid sequences of E-cadherin were translated by Snapgene Viewer. Numbers, amino acid sequence.

To assess whether BFT can induce E-cadherin cleavage in mouse epithelial cell lines, five mouse cell lines were treated with the serum-free medium (SFM), rNTBF, or rETBF supernatant for 3 h and analyzed by Western blotting. The level of the full-length E-cadherin (120 kDa) in MSIE, CT26, YAMC, and B16 cells remained unchanged after treatment with the rETBF (i.e., active BFT) supernatant (1:20 dilution) (Figure 1a). In contrast, full-length E-cadherin levels in CMT93 cells disappeared after 3 h in response to rETBF treatment. These results suggest that CMT93 cells are responsive to BFT treatment. This result was confirmed using immunofluorescence confocal microscopy in CMT93 cells. The complete loss of the E-cadherin signal was seen in the CMT93 cells treated with rETBF (Figure 1b). The rNTBF supernatant (i.e., inactive BFT) showed no effect and was comparable to the serum-free media control, suggesting that catalytically active BFT is required for biological activity in mouse cells. A hallmark of BFT biological activity is the induction of cell rounding due to the loss of E-cadherin, which binds adjacent epithelial cells. As expected, rETBF promoted cell rounding in CMT93 cells, whereas rNTBF showed no morphologic changes, similar to the serum-free media control (Figure 1c).

Since BFT is structurally similar to matrix metalloproteinase (MMP), the predicted BFT-induced E-cadherin cleavage site is thought to lie between isoleucine (700) and valine (701), near the extracellular region next to the cell membrane (Figure 1d, red). To investigate if the susceptibility to BFT-induced E-cadherin cleavage was due to amino acid differences in the putative E-cadherin cleavage site, we sequenced the region encompassing the putative cleavage site and compared the amino acid sequences between BFT-responsive (CMT93) and BFT-nonresponsive cell lines (MSIE and YAMC) (Figure 1d). We found no differences in the amino acid sequences, suggesting that the E-cadherin amino sequence is not responsible for BFT responsiveness.

### 3.2. BFT Induces E-Cadherin Ectodomain Cleavage and Degradation of Intracellular Domain in CMT93 Cells

**Figure 2:**
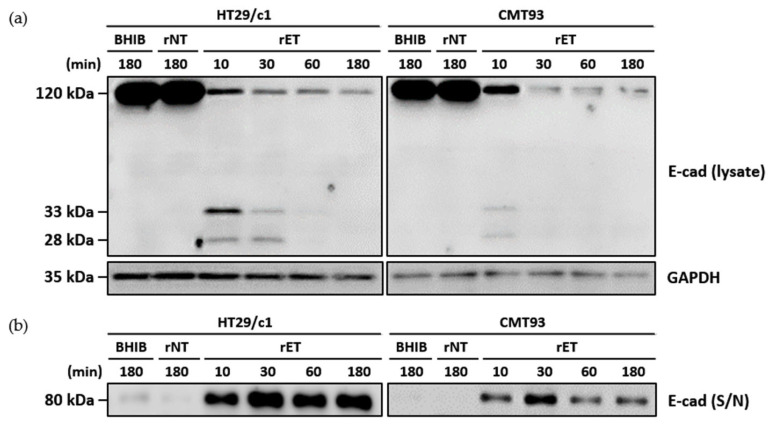
BFT induces degradation of intracellular domain after E-cadherin ectodomain cleavage in CMT93 cells. (**a**) HT29/c1 and CMT93 cells were treated with brain heart infusion broth (BHIB), rNTBF, or rETBF supernatant (1:20 dilution) for indicated times (10, 30, 60, and 180 min). Cell lysates of HT29/c1 and CMT93 cells were examined by Western blotting using anti-E-cadherin endodomain antibody (clone C36). GAPDH, internal control; (**b**) Cell culture media from BFT-treated HT29/c1 and CMT93 cells were concentrated using centrifugal filters and examined by Western blotting, using a polyclonal anti-E-cadherin ectodomain antibody (clone H108).

To determine whether the murine cell line CMT93 undergoes E-cadherin cleavage and the subsequent degradation of the intracellular domain similar to the human cell line HT29/c1, cells were treated with the rNTBF or rETBF supernatant for different times and assessed by Western blot analysis (Figure 2a). Using the anti-E-cadherin endodomain-specific antibody, the 120 kDa full-length E-cadherin band rapidly decreased within 10 min in both cell lines. Concomitantly, the 33 kDa intracellular domain and 28 kDa cytoplasmic fragment of E-cadherin were observed at 10 min. The 33 kDa and 28 kDa cleavage products underwent further degradation and were undetectable at later time points. Using the anti-E-cadherin ectodomain-specific antibody, the “shed” 80 kDa fragment was detected in the cell culture media (Figure 2b). Since the metabolic half-life of E-cadherin is approximately 5–10 h [23] and because the BFT-induced E-cadherin cleavage occurred rapidly, the sudden decrease in full-length E-cadherin is most likely not as a result of decreased E-cadherin synthesis but the cleavage of the full-length E-cadherin. Taken together, the E-cadherin cleavage process in CMT93 cells appears to be identical to that in HT29/c1 cells.

### 3.3. CMT93 Cells Resynthesize E-Cadherin and Undergo Proliferation After BFT Treatment

**Figure 3:**
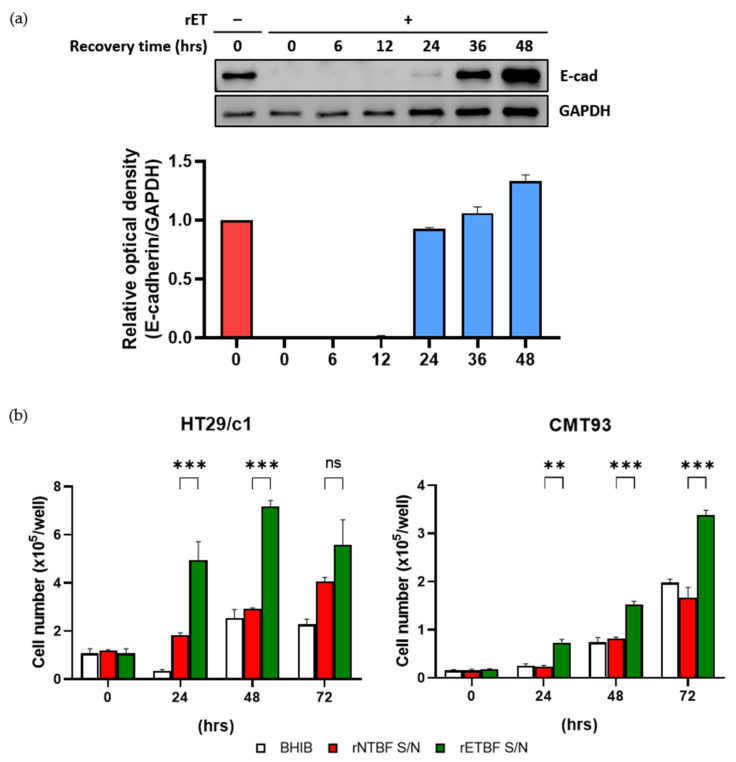
(**a**) CMT93 cells were treated with rETBF supernatant (1:20 dilution) for 3 h, washed, and replaced with complete medium. Western blot of E-cadherin changes over time after 3 h of rETBF supernatant treatment. Cell lysates of CMT93 cells were examined via Western blotting. Relative E-cadherin band intensity was quantified using FusionCapt Advance software (version 16.06). Western blot densitometry results are shown as the mean and standard error of the mean (SEM) of three independent experiments. GAPDH, internal control. Red, non-treated cells; Blue, rETBF-treated cells; (**b**) HT29/c1 (left) and CMT93 (right) cells were treated with BHIB, rNTBF, or rETBF supernatant and then enumerated using a hemacytometer. The cell numbers were assessed by three independent experiments and are shown as the mean and SEM. The results were evaluated with Student’s *t*-test. ** *p* < 0.01; *** *p* < 0.001; ns, not significant.

It has been reported that E-cadherin resynthesis begin several hours after BFT-induced cleavage in HT29/c1 cells [24]. Furthermore, the removal of BFT leads to HT29/c1 cells recovering a normal cell morphology within 2–3 days after BFT treatment [25,26]. To investigate whether CMT93 cells resynthesize E-cadherin after BFT treatment, CMT93 cells were treated with the rETBF supernatant for 3 h, and the BFT was subsequently removed by repeated washing. Thereafter, CMT93 cells were incubated for the indicated time periods (6, 12, 24, 36, and 48 h), and the cell lysates were examined by Western blotting using the endodomain-specific antibody (Figure 3a). Consistent with HT29/c1, CMT93 cells lost their E-cadherin by 3 h after BFT treatment, and the levels of the full-length E-cadherin began to appear after 24 h and gradually returned to normal by 36 h. This result indicates that the recovery of E-cadherin in BFT-treated CMT93 cells is similar to that of HT29/c1 cells.

Next, to determine if BFT stimulates proliferation in CMT93 cells, cells were seeded at a density of 5 × 10^4^ cells/well and treated with BHIB, rNTBF, or rETBF supernatant (1:20 dilution) for the indicated time periods (0, 24, 48, and 72 h), and the viable cells were counted using a hemacytometer (Figure 3b). Similar with HT29/c1, the number of CMT93 cells significantly increased compared to the control groups (BHIB and rNTBF) after BFT treatment. These results indicate that the BFT-treated CMT93 cells resynthesize E-cadherin and undergo cellular proliferation similar to BFT-treated HT29/c1 cells.

### 3.4. γ-Secretase Cleaves E-Cadherin Intracellular Domain

**Figure 4:**
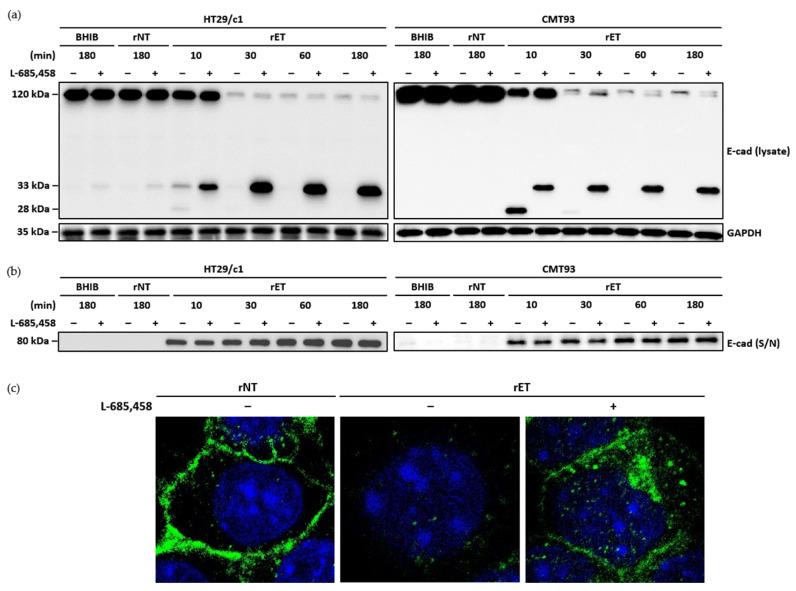
Inhibition of γ-secretase blocks BFT-induced cleavage of intracellular domain of E-cadherin in CMT93 cells. (**a**) HT29/c1 and CMT93 cells were preincubated with γ-secretase inhibitor L-685,458 (1.5 μM) for 60 min and treated with BHIB, rNTBF, or rETBF supernatant (1:20 dilution) for indicated time periods (10, 30, 60, and 180 min). Cell lysates of HT29/c1 and CMT93 cells were examined by Western blot analysis using anti-E-cadherin endodomain antibody (C36); (**b**) Presence of shed E-cadherin was detected in cell culture supernatant (S/N) using anti-E-cadherin ectodomain-specific antibody (H108). GAPDH, internal control; (**c**) Immunofluorescence imaging. CMT93 cells were preincubated with γ-secretase inhibitor L-685,458 (1.5 μM) for 60 min and treated with rNTBF or rETBF supernatant for 180 min. Images of E-cadherin (green) and nuclei (blue) were taken with a confocal laser scanning microscope (×1600).

In BFT-treated HT29/c1 cells, the 33 kDa membrane-tethered intracellular domain is subsequently cleaved by γ-secretase. To investigate whether in CMT93 cells, γ-secretase cleaves the 33 kDa intracellular domain after full-length E-cadherin cleavage induced by BFT, the CMT93 cells were preincubated with the γ-secretase inhibitor (L-685,458), then treated with the BHIB, rNTBF, or rETBF supernatant (Figure 4a). The Western blot analysis showed that in the CMT93 cells pretreated with the γ-secretase inhibitor, the 33 kDa intracellular domain accumulated, whereas the treatment without the γ-secretase inhibitor resulted in the rapid degradation of the 33 kDa intracellular domain and 28 kDa fragment. As expected, the ectodomain cleavage of E-cadherin was unaffected by the γ-secretase inhibitor (Figure 4b). To determine whether the 33 kDa intracellular domain was tethered to the cell membrane after BFT treatment, the CMT93 cells were examined using immunofluorescence confocal microscopy using the E-cadherin endodomain-specific antibody (Figure 4c). After 180 min of treatment with the rETBF supernatant, the E-cadherin signal was still localized at the cell membrane in cells pretreated with the γ-secretase inhibitor. In contrast, the E-cadherin signal was undetectable in cells not treated with the γ-secretase inhibitor. Taken together, γ-secretase is essential for the subsequent cleavage of the intracellular domain in the CMT93 cell line.

### 3.5. Proteasome Degrades E-Cadherin Cytoplasmic Fragment in CMT93 Cells

**Figure 5:**
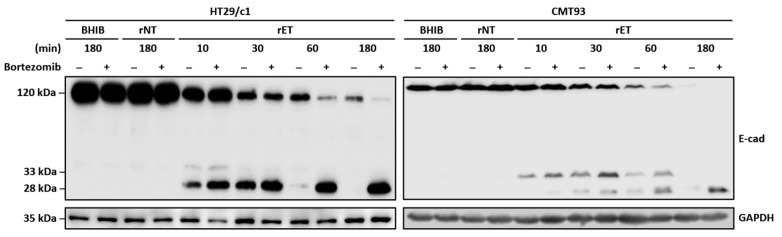
BFT induces degradation of 28 kDa cytoplasmic fragment in HT29/c1 and CMT93 cells. HT29/c1 and CMT93 cells were preincubated with proteasome inhibitor bortezomib (2 μM) for 60 min and treated with BHIB, rNTBF, or rETBF supernatant for indicated time periods (10, 30, 60, and 180 min). Cell lysates were examined by Western blot analysis using anti-E-cadherin endodomain antibody (C36). GAPDH, internal control.

Nonfunctional cellular proteins are subsequently degraded by the proteasome [27]. To determine whether the proteasome complex degrades the 28 kDa E-cadherin cytoplasmic fragment generated after the 33 kDa intracellular domain cleavage by γ-secretase, CMT93 cells were preincubated with a proteasome inhibitor (bortezomib) and treated with the BHIB, rNTBF, or rETBF supernatant (Figure 5). The Western blot analysis showed that CMT93 cells treated with the proteasome inhibitor accumulated the 28 kDa cytoplasmic fragment, whereas the cells not treated with the proteasome inhibitor showed a degradation of the 28 kDa fragment. These data suggest that, similar to HT29/c1 cells, the 28 kDa cytoplasmic fragment in CMT93 cells is degraded by the proteasome complex.

### 3.6. BFT Induces Secretion of CXCL1 and CXCL5

**Figure 6:**
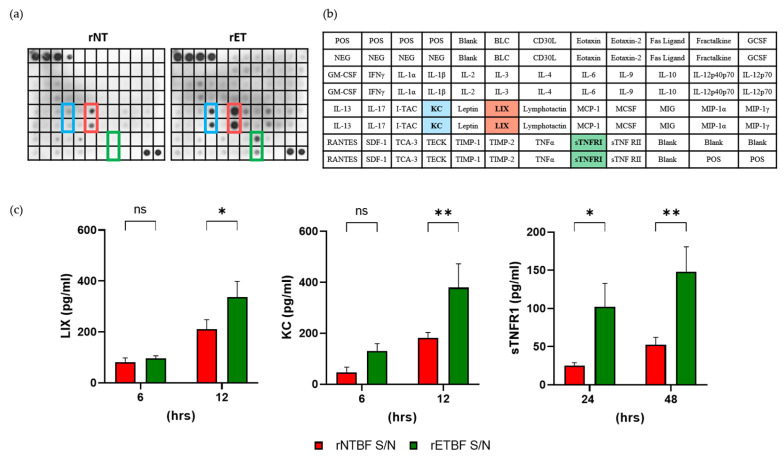
BFT increases formation of KC, LIX, and sTNFR1 in CMT93 cells. (**a**) Spectrum of inflammatory proteins secreted in vitro from CMT93 cells. Cells were treated with rNTBF or rETBF (1:20 dilution) for 24 h and cell culture media incubated with the inflammation array membrane. Bound proteins were visualized using ECL. Scanned images are shown. Blue, KC; Red, LIX; Green, sTNFR I; (**b**) The position of antibodies and list of the target proteins assessed in the membrane array. Blue, KC; Red, LIX; Green, sTNFR I; (**c**) CMT93 cells were treated with rNTBF or rETBF supernatant, and levels of LIX, KC, and sTNFR1 in cell culture media were assessed by ELISA. The samples were analyzed by three independent experiments and are shown as the mean and SEM. The results were evaluated with Student’s *t*-test. * *p* < 0.05; ** *p* < 0.01; ns, not significant.

It has been reported that BFT induces the secretion of IL-8 inflammatory cytokine in the HT29/c1 human epithelial cell line [28]. To determine if BFT induces the secretion of inflammatory cytokines, as well as other proteins, CMT93 cells were treated with rNTBF or rETBF, and the cell culture medium was analyzed using an inflammation antibody array. Two cytokines, keratinocyte chemoattractant (KC) and lipopolysaccharide-induced CXC chemokine (LIX), were detected at higher levels in the rETBF-supernatant-treated CMT93 cells compared to the rNTBF-supernatant-treated cells (Figure 6a,b). In addition, the soluble TNF-α receptor 1 (sTNFR1) level was also elevated in the rETBF-treated CMT93 cells. To confirm these results, an ELISA was performed to detect KC, LIX, and sTNFR1 levels (Figure 6c). The results indicate the levels of KC and LIX significantly increased after 12 h of the rETBF supernatant treatment. The sTNFR1 level significantly increased by 24 h. These results indicate that BFT induces the generation of three proteins in CMT93 cells.

## 4. Discussion

ETBF is a pro-carcinogenic bacterium that promotes intestinal barrier permeability and DNA damage in intestinal epithelial cell monolayers, which can contribute to colitis-associated cancer [29,30]. The cleavage of the adherence junction protein E-cadherin results in β-catenin-dependent nuclear signaling, the further induction of the proto-oncogene protein c-Myc, and the proliferation and secretion of the proinflammatory chemokine IL-8. All of these effects contribute to the colonic inflammatory response observed in ETBF-infected animals [14,24]. Mechanistic studies regarding BFT-induced E-cadherin cleavage have been extensively conducted in human intestinal epithelial cell lines [31]. Previously, we showed that BFT induced the E-cadherin ectodomain cleavage of mouse intestinal tissues ex vivo, suggesting that both human and mouse epithelial cells respond similarly to BFT [12]. Until now, the lack of a BFT-responsive mouse epithelial cell hindered in-depth cellular studies in mice. In the current study, we identified the CMT93 cell line as a BFT-responsive mouse intestinal epithelial cell line that shows identical responses to BFT in human cell lines.

CMT93 cells showed many similarities with the HT29/c1 cell line in response to BFT treatment, such as cell rounding, E-cadherin resynthesis, and cell proliferation. It has been reported that BFT induces the cell proliferation activation of the β-catenin/TCF signaling pathway and c-myc expression [14]. We predict that similar events are occurring in BFT-treated CMT93 cells. The identification of the BFT-responsive cell line will be instrumental in these types of studies. BFT is proposed to bind to a putative host cell receptor, which then leads to the subsequent cleavage of E-cadherin and the abovementioned downstream events [10]. Having identified both BFT-responsive and BFT-nonresponsive mouse cell lines, we hypothesize that the BFT-nonresponsive mouse cell lines lack this putative cell receptor. We can now undertake this line of research using the cell lines shown in the current study.

BFT induces the secretion of IL-8 in HT29/c1 cells. Previously, we showed that large intestinal tissues from ETBF-infected mice secrete several proteins (G-CSF, IL-6, IL-17, KC, LIX, MIP-1γ, and MCP-1) ex vivo [32]. LIX, also known as chemokine C-X-C motif ligand 5 (CXCL5), is an epithelial-derived neutrophil-activating peptide belonging to the CXC chemokine family and is produced in mice. LIX is produced following the stimulation of cells with the interleukin-1 (IL-1) or TNF-α inflammatory cytokines [33]. KC, also known as chemokine C-X-C motif ligand 1 (CXCL1), is a small peptide belonging to the CXC chemokine family that can be produced by epithelial cells, macrophages, and neutrophils in mice. KC is a functional homologue of IL-8 that acts as a chemoattractant for immune cells, especially neutrophils to the injured or infected sites [19]. However, the ex vivo tissues contained not only epithelial cells but other cell types, including immune cells. Therefore, it was unclear whether the source of these cytokines was from epithelial cells and/or other cell types. Furthermore, the secretion LIX and KC may be due to an indirect activation by other cytokines from immune cells. In the current study, we found that KC and LIX secretion was induced by active BFT in CMT93, implicating that these two cytokines were directly secreted from epithelial cells.

To elucidate how LIX and KC secretion were induced by BFT in CMT93 cells, further studies are necessary. Previous studies have reported that BFT induces the nuclear translocation of β-catenin, leading to increased cellular proliferation and IL-8 secretion in the HT29/c1 cell line [14]. β-catenin is a transcription factor that is normally degraded by the APC/axin/GSK-3β complex [34,35]. However, β-catenin is also found to bind to the cytoplasmic domain of E-cadherin, and BFT-induced E-cadherin cleavage can result in the translocation of β-catenin to the nucleus [17]. The translocated β-catenin can activate IL-8 expression [36]. We previously showed that the treatment of HT29/c1 cells with LiCl, which activates the β-catenin pathway, can induce IL-8 secretion [37]. In addition, we previously generated β-catenin-deficient HT29/c1 cells using the CRISPR-Cas9 method [15]. The secretion of IL-8 was abrogated in β-catenin-deficient HT29/c1 cells treated with BFT. To determine whether the β-catenin pathway is required for the BFT-induced expression of KC and LIX, siRNA knockdown and/or knockout studies using CRISPR-Cas9 methods will be necessary.

In the results obtained from the inflammation antibody array and ELISA, the rETBF supernatant increased the sTNFR1 levels. TNFR1 is the receptor of TNF-α, and sTNFR1 is the soluble cleaved form of TNFR1. It has been reported that the proteolysis of the ectodomain of several membrane-tethered proteins is activated in cancer cells [38]. In colonic epithelial cancer cells, the soluble TNF-α receptor has been found to be released into cell culture supernatants through metalloprotease-mediated proteolytic cleavage. sTNFR1 levels statistically increased in the rETBF-treated CMT93 cells at 24 h. We generally do not examine protein cleavage after 12 h, and thus, it is possible that sTNFR1 may be cleaved by BFT at a later time point. This raises the possibility that the host cell proteases responsible for TNFR1 cleavage may be different from proteases involved in E-cadherin cleavage. Further confirmative studies need to be performed in both HT29/c1 cells and CMT93 cells using TNFR1 ectodomain- and endodomain-specific antibodies.

The main objective of the current study was to determine if BFT can exert biological activity in a murine intestinal cell line similar to that in human intestinal cell lines. The identification of the BFT-responsive murine CMT93 cell line implicates that BFT’s mode of action is not species-specific. However, two major limitations in the current study exist. The first is that we have yet to provide any novel insights into BFT function. Our current hypothesis, based on various indirect pieces of evidence and negative data, is that BFT does not directly bind and cleave E-cadherin. We favor the hypothesis that BFT binds to an unidentified host cell receptor, which promotes a signaling cascade, which in turn, activates host protease(s) that directly cleave E-cadherin. The identification of a BFT-responsive cell line (i.e., CMT93) and BFT-nonresponsive cell lines will allow us to possibly conduct screening (e.g., microarrays) to identify potential host receptors. The premise is that the BFT-nonresponsive cell lines may lack the host cell receptor and/or the downstream host protease. The second limitation is that we did not use purified BFT with a known concentration. We have faced technical difficulties and have yet to succeed in isolating purified BFT in a sufficient quantity. We are currently generating bacteria strains expressing his-tagged BFT to overcome this problem. The utilization of recombinant BFT will be necessary for future mechanistic studies.

In conclusion, we have identified a BFT-responsive mouse intestinal cell line that reacts to BFT similarly to human epithelial cell lines. The additional identification of BFT-nonresponsive cell lines should enhance further research in both the identification of the putative cell receptor as well as the understanding of BFT’s mode of action in ETBF infection mouse models.

## 5. Conclusions

In this study, we report for the first time a BFT-responsive murine cell line (CMT93) showing that BFT induces E-cadherin ectodomain cleavage and the secretion of KC (CXCL1) and LIX (CXCL5). The E-cadherin ectodomain cleavage by BFT suggests no amino acid sequence differences between BFT-responsive and BFT non-responsive mouse epithelial cells. The cytoplasmic cleavage of E-cadherin is dependent on γ-secretase, and subsequent degradation occurs via the proteasome complex. These results suggest that the BFT-induced cellular responses are conserved in both humans and mice. In addition, the BFT-induced effects verified in the mouse cell line will provide a mechanistic understanding for investigating ETBF effects in the murine model.

## Data Availability

The original contributions presented in this study are included in the article/Supplementary Material. Further inquiries can be directed to the corresponding author (K.-J.R.: kjrhee@yonsei.ac.kr).

## Supplementary Materials

The following supporting information can be downloaded at: https://www.mdpi.com/article/10.3390/microorganisms13040781/s1, Table S1: Position and names of inflammatory mediators in antibody array.

## Abbreviations

The following abbreviations are used in this manuscript:

APC	Adenomatous polyposis coli
BFT	*Bacteroides fragilis* toxin
BHIA	Brain heart infusion agar
BHIB	Brain heart infusion broth
BSA	Bovine serum albumin
cDNA	Complementary deoxyribose nucleic acid
ELISA	Enzyme-linked immunosorbent assay
ETBF	Enterotoxigenic *Bacteroides fragilis*
GAPDH	Glyceraldehyde-3-phosphate dehydrogenase
G-CSF	Granulocyte-colony stimulating factor
GSK-3β	Glycogen synthase kinase-3 beta
IL	Interleukin
KC	Keratinocyte chemoattractant
LIX	Lipopolysaccharide-induced CXC chemokine
MCP-1	Monocyte chemoattractant protein-1
MIP-1γ	Macrophage inflammatory protein-1 gamma
MMP	Matrix metalloprotease
mRNA	Messenger ribose nucleic acid
NF-κB	Nuclear factor-kappa-light-chain-enhancer of activated B cells
NTBF	Non-toxigenic *Bacteroides fragilis*
rETBF	Recombinant enterotoxigenic *Bacteroides fragilis*
rNTBF	Recombinant non-toxigenic *Bacteroides fragilis*
S/N	Supernatant
SEM	Standard error of the mean
SFM	Serum-free medium
STAT3	Signal transducer and activator of transcription 3
sTNFR1	Soluble tumor necrosis factor receptor 1
TGF-β	Transforming growth factor-beta
Th17	T helper 17
TNF-α	Tumor necrosis factor-alpha
