# Supplementary material for: Bacteroides fragilis Toxin Induces Sequential Proteolysis of E-Cadherin and Inflammatory Response in Mouse Intestinal Epithelial Cell Line
Source: Microorganisms. 2025 Mar 28;13(4):781. doi: 10.3390/microorganisms13040781 (PMC12029241; doi:10.3390/microorganisms13040781)
Supplement: Supplementary file 1 [file microorganisms-13-00781-s001.zip › microorganisms-3525562-supplementary.pdf]

Table S1. Position and names of inflammatory mediators in antibody array.

Position	Abbreviation	Full name	Other names
A1-A4, H11, H12	POS	Positive control	-
A5, B5, G10-G12, H10	Blank	Blank	-
A6, B6	BLC	B-lymphocyte chemoattractant	
A7, B7	CD30L	CD30 ligand	
A8, B8	Eotaxin	Eotaxin	CCL11
A9, B9	Eotaxin-2	Eotaxin-2	CCL24
A10, B10	Fas Ligand	Fas Ligand	-
A11, B11	Fractalkine	Fractalkine	CX3CL1
A12, B12	GCSF	Granulocyte colony-stimulating factor	-
B1-B4	NEG	Negative control	-
C1, D1	GM-CSF	Granulocyte-macrophage colony-stimulating factor	-
C2, D2	IFNγ	Interferon-gamma	-
C3, D3	IL-1α	Interleukin-1 alpha	-
C4, D4	IL-1β	Interleukin-1 beta	-
C5, D5	IL-2	Interleukin-2	-
C6, D6	IL-3	Interleukin-3	-
C7, D7	IL-4	Interleukin-4	-
C8, D8	IL-6	Interleukin-6	-
C9, D9	IL-9	Interleukin-9	-
C10, D10	IL-10	Interleukin-10	-
C11, D11	IL-12p40p70	Interleukin-12p40p70	-
C12, D12	IL-12p70	Interleukin-12p70	-
E1, F1	IL-13	Interleukin-13	-
E2, F2	IL-17	Interleukin-17	-
E3, F3	I-TAC	Interferon-inducible T-cell alpha chemoattractant	CXCL11
E4, F4	KC	Keratinocyte-derived chemokine	CXCL1
E5, F5	Leptin	Leptin	-
E6, F6	LIX	LPS-induced CXC chemokine	CXCL5
E7, F7	Lymphotactin	Lymphotactin	-
E8, F8	MCP-1	Monocyte chemoattractant protein-1	CCL2
E9, F9	MCSF	Macrophage colony-stimulating factor	-
E10, F10	MIG	Monokine induced by gamma interferon	CXCL9
E11, F11	MIP-1α	Macrophage inflammatory protein-1 alpha	CCL3
E12, F12	MIP-1γ	Macrophage inflammatory protein-1 gamma	CCL9
G1, H1	RANTES	Regulated upon activation, normal T-cell expressed and secreted	CCL5
G2, H2	SDF-1	Stromal cell-derived factor-1	CXCL12
G3, H3	TCA-3	T-cell activation gene 3 protein	CCL1
G4, H4	TECK	Thymus-expressed chemokine	CCL25
G5, H5	TIMP-1	Tissue inhibitor of metalloproteinases-1	-
G6, H6	TIMP-2	Tissue inhibitor of metalloproteinases-2	-
G7, H7	TNFα	Tumor necrosis factor-alpha	-
G8, H8	sTNFR I	Soluble tumor necrosis factor receptor I	-
G9, H9	sTNFR II	Soluble tumor necrosis factor receptor II	-
